# kV reference dosimetry in Australia and New Zealand: Survey results and trends

**DOI:** 10.1002/acm2.14458

**Published:** 2024-07-18

**Authors:** Madelaine Tyler, Mitchell Duncan, Joanne McNamara

**Affiliations:** ^1^ Shoalhaven Cancer Care Centre Nowra New South Wales Australia; ^2^ Townsville Cancer Centre Townsville Queensland Australia

**Keywords:** dosimetry, kilovoltage, radiotherapy

## Abstract

**Purpose:**

To assess the number of radiotherapy kilovoltage (kV) units in service, their clinical utilization, and methodology and equipment used for absorbed dose determination across Australia and New Zealand.

**Methods:**

A survey was sent to 61 Australian and New Zealand radiotherapy providers in the second half of 2023.

**Results:**

Fifty‐seven responses were received, with 43 departments having kV units and providing beam quality data for 185 therapeutic kV beams 20–300 kVp. Percentage depth dose curves were compared between five clinical beams with 100 kVp and 2.13–6.28 mm Aluminum half value layers (HVLs), demonstrating large differences that can occur between beams with the same kVp. Eighteen departments provided clinical utilization data for their kV units, with a total of 4458 treatment courses and their corresponding kVp reported. All departments complied with national and international recommendations with respect to the equipment used for reference dosimetry of kV beams; 77% of ionization chambers used for absorbed dose determination were of Farmer‐type, with the remaining 23% being plane parallel soft x‐ray chambers. Methods of derivation of air‐kerma calibration factors varied, with 73% of respondents using a draft document disseminated by the Australian Primary Standards laboratory, 23% using HVL alone, and 6% using other methods.

**Conclusions:**

The results of this survey provide a snapshot of kilovoltage radiation therapy use and the number of kV units across Australia and New Zealand. This data can be used as a point of reference for future investigations into clinical utilization and reference dosimetry methods across Australia and New Zealand or for comparisons with other countries, facilitating standardization of reference dosimetry practice for kilovoltage units.

## 1 INTRODUCTION

In Australia and New Zealand, skin cancers including melanoma and non‐melanoma were estimated to account for 37.3% of new cancer diagnoses in 2020. This figure represents the highest incidence globally, surpassing that of any other nation by twofold.^1^ It is anticipated that the actual incidence rates are higher than officially reported, as basal cell carcinoma and squamous cell carcinoma of the skin are not notifiable diseases in Australia. Consequently, reported case numbers are derived solely from specific procedures documented in Medicare billing records.^2^

Skin cancer and benign skin conditions are often treated using low energy kilovoltage (kV) x‐rays.^3^, ^4^, ^5^ Many radiotherapy centers are equipped with kV therapy units generating x‐ray beams up to 300 kVp with typical half‐value layer (HVL) ranges between 0.035 mm Aluminum to 3 mm Copper.^6^, ^7^

In 2018, the Australasian College of Physical Scientists and Engineers in Medicine (ACPSEM), the guiding body for radiation oncology medical physics practice in Australia and New Zealand, published recommendations for quality assurance in kilovoltage radiotherapy. These recommendations included specifications on methods and equipment for use in reference dosimetry.^4^ The publication states that reference dosimetry measurements should be performed using the American Association of Physicists in Medicine (AAPM) Task Group 61 protocol for 40–300 kV x‐ray beam dosimetry in radiotherapy and radiobiology.^8^ AAPM TG61 is recommended as it is based on air‐kerma free in air (K_air_) standards which are provided by the Australian primary standards dosimetry laboratory. Ionization chamber calibration is required to be traceable back to the Australian or New Zealand primary standards for K_air._ ^4^

The Australian Radiation Protection and Nuclear Safety Agency (ARPANSA) is the primary standards laboratory used for radiotherapy instrument calibration in Australia. Dosimeters used for reference dosimetry of kV beams are calibrated in the low energy x‐ray (LEX) and/or medium energy x‐ray (MEX) beam sets in terms of K_air_. This results in an air‐kerma calibration factor (N_K_) for the dosimeter, which varies as a function of beam quality. Details on the Australian primary standards for air‐kerma have been published by ARPANSA in 2022.^9^

The LEX calibration service provides direct calibration of an ionization chamber with the primary standard for low energy x‐rays, the Low Energy Free Air Chamber, in a set of eight beams with generating potentials (kVp) between 20 and 100 kVp (0.11–6.53 mm Al HVL). The LEX calibration service is designed for thin‐window plane‐parallel ionization chambers but can also be utilized for Farmer‐type cylindrical ionization chambers.

The MEX calibration service provides direct calibration of an ionization chamber with the primary standard for medium energy x‐rays, the Medium Energy Free Air Chamber, in a set of kV beams with kVp ranging between 40 and 320 kVp (0.57 mm Al–4.34 mm Cu HVL). Varying amounts of filtration are added to a specific kVp to provide a set of 59 individual beam qualities with the thickness of added filtration denoted by a discrete beam code (Table 1). Each kVp has several added filtration combinations to modify the beam quality, for example, there are five beams with nominal kVp = 100 kV with various aluminum filtration corresponding to codes J, K, B, C, and D. This results in five unique beam qualities for 100 kVp denoted as NX**K**100, NX**J**100, NX**B**100, NX**C**100, and NX**D**100. These beams have corresponding HVLs of 1.30, 2.05, 4.74, 5.49, and 6.61 mm Al. The MEX calibration service is designed for use with Farmer‐type cylindrical ionization chambers.

**TABLE 1:** Beam filtration codes and associated thicknesses for the ARPANSA MEX calibration beam set.

	Added filtration	
MEX beam code	mm Al	mm Cu
A	4.0	
B	4.5	
C	6.0	
D	9.0	
E	4.0	0.5
F	4.0	1.0
G	4.0	1.6
H	4.0	3.0
I	4.0	5.0
J	0.5	
K	1.0	

The ACPSEM^4^ and AAPM TG61^8^ recommend interpolation of ionization chamber N_K_ in terms of both kVp and HVL as the beam qualities for the user kV unit will not always match that of the standards laboratory. A draft document published by ARPANSA in 2017 titled “*Suggested method for interpolation from the ARPANSA MEX beam set to a clinical beam Q, for a Farmer‐type chamber*” outlines the recommended methods of interpolation for ionization chamber N_K_ in the MEX dataset in the user beam quality (HVL_Q_).

The method of N_K_ derivation for an ionization chamber using the ARPANSA document depends on the user kVp/HVL. Four Cases/Scenarios are provided with instructions for each:

Case 1: There is a matching kVp in the ARPANSA beams and HVL_Q_ is bounded by the lowest and highest HVL in this set of ARPANSA beams. In this case, it is recommended to interpolate in the matching kVp of the clinical beam between the MEX HVLs that bracket the clinical beam HVL.
Case 2: There is a matching kVp in the ARPANSA beams and HVL_Q_ is above the highest or below the lowest HVL in the ARPANSA set. This is similar to Case 1, but extrapolation is performed using two MEX beams with HVL closest to the clinical beam HVL.
Case 3: There are no matching kVp beams in the ARPANSA set. It is recommended to choose kVp above and below the clinical beam kVp, perform interpolation or extrapolation as per Cases 1 and 2 for these beams to obtain estimates of the N_K._ Linear interpolation using kVp is then performed to find the N_K_ for the user beam.
Case 4: Interpolation with fixed filtration. This case is an alternative to Case 1−3. The user will group the MEX beams according to the filtration (as shown in Table 1). An interpolation is then performed between the MEX HVLs that bracket the clinical beam HVL.

There are no published mandatory methods of interpolation of chamber N_K_ data as standard laboratories in various regions provide calibration sets that are quite different in configuration. For example, ARPANSA provides up to five beams with the same kVp and different filtration, whereas the National Institute of Standards and Technology provides beams with low, medium, and high additional filtration.^10^ The Nk values provided by the standards laboratory and the local method of interpolation between these Nk values directly impact the dose calibration of the kilovoltage unit.

The most recent data on the number of kV units in Australia was published over a decade ago, in 2010, with 26 kV units reported.^3^ Kilovoltage x‐rays beam quality is specified in terms of kVp and HVL.^4^, ^8^ The 2010 survey reported 183 kV beams, with 37 different peak voltages. For each kVp, beams had differing HVLs, resulting in a wide range of beam qualities for single‐generating potentials. Variation in penetrative quality for a single kVp means that treatment outcomes cannot be equated between different centers or across units for a single kVp. It is crucial that oncologists are aware of the dose distribution achievable with their own unit to effectively treat patients.

No published data exists for the number of kV units in New Zealand. Details of kilovoltage unit reference dosimetry and methods employed to obtain N_K_ for dosimetry equipment in the clinics in Australia and New Zealand have not been previously published.

This manuscript reports on the results of a survey distributed to Australian and New Zealand radiotherapy departments in late 2023 to assess the clinical utilization of kV units and collate information on common methods and equipment utilized for reference dosimetry. It is anticipated that this study may stimulate similar audits worldwide and facilitate the standardization of kilovoltage reference dosimetry internationally.

## 2 METHODS

A short survey was designed to assess the number of radiotherapy kV units across Australia and New Zealand, their clinical utilization, and the methodology and equipment used for absorbed dose determination. Both private and public service providers were contacted with a 1‐month timeframe for completion requested. Where no response was received, additional effort was made to contact the relevant staff and department.

The survey was distributed through email correspondence in the second half of 2023 to the medical physics directors and state/territory leads in Australian and New Zealand radiotherapy centers, totaling 61. Within Australia and New Zealand, some radiotherapy providers have multiple centers, utilizing equipment with similar characteristics (or sharing equipment) and have uniform protocols across multiple sites. In this instance, the site lead for each state or territory was contacted requesting completion of the survey, not individual centers.

Responses were collated, and a summary of the results was sent to all respondents in late 2023. Analysis of survey results was based on the number of individual responses received and is not indicative of the total number of therapeutic kV units providing radiotherapy in Australia and New Zealand.

The survey was comprised of the following questions:

1. What are your clinical beam qualities? (Kilovoltage generating potential: kVp/Half Value Layer).
2. What chamber type/model do you send to ARPANSA for calibration in kV beams?
3. What is the method used to obtain N_K_ (Air KERMA Calibration factor) in your clinical beam from the ARPANSA MEX data provided?
4. If you were able to provide further information on the clinical utilization of your beams (i.e., number of patients treated with xx kVp) over the last 5 years it would assist in generating a deeper understanding of the clinical impact of N_K_ derivation methods.

## 3 RESULTS

A total of 57 responses to the survey were received, equating to a 93% response rate. Of those respondents, 43 had kV units and were able to provide data for 185 therapeutic kV beams.

### 3.1 Beam quality

**FIGURE 1:**
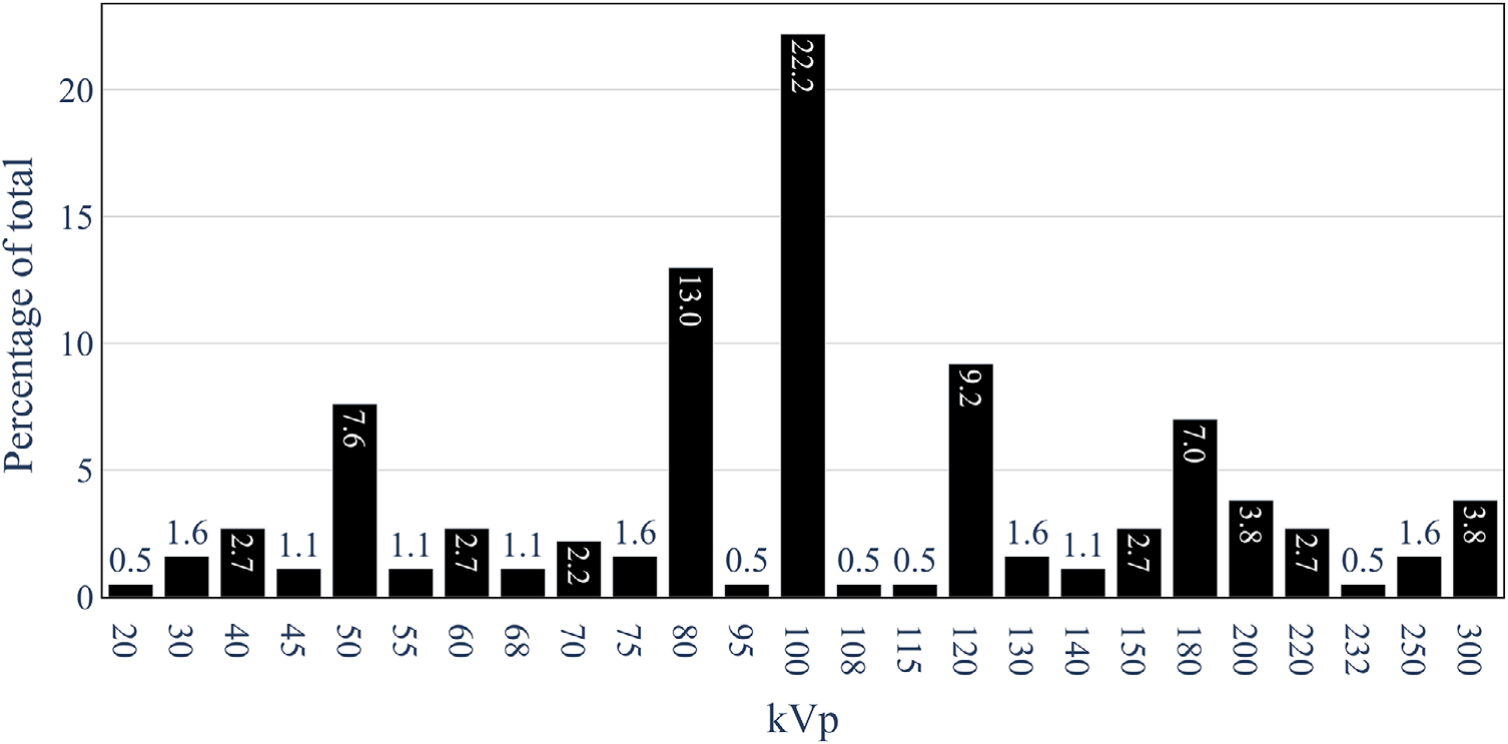
Relative frequency distribution of beam kVp for 185 therapeutic beams reported in the survey across Australia and New Zealand.

Figure 1 is a relative frequency histogram of all beam‐generating kV potentials reported in response to question 1. The median kVp across centers were 100 kVp (22.2% with 26 different peak voltages ranging from 20 to 300 kVp reported).

**FIGURE 2:**
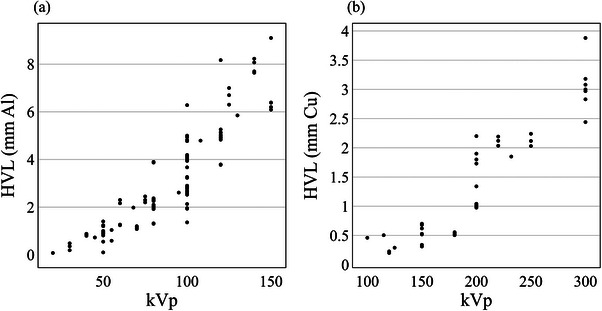
Half value layer of Aluminum (a) and Copper (b) as a function of kV generating potential (kVp) for beams reported in the survey across Australia and New Zealand.

There was a large distribution in kV beam qualities (in terms of kVp and HVL) in clinical use across Australia and New Zealand. The distribution of HVL as a function of the kVp for beam qualities defined by (a) Aluminum and (b) Copper half‐value layers is shown in Figure 2. A broad range of HVLs was reported for each kVp, with the largest range found at 100 kVp (1.36–6.20 mm Aluminum) and 200 kVp (0.95–2.20 mm Copper), for Aluminum and Copper HVLs, respectively.

**FIGURE 3:**
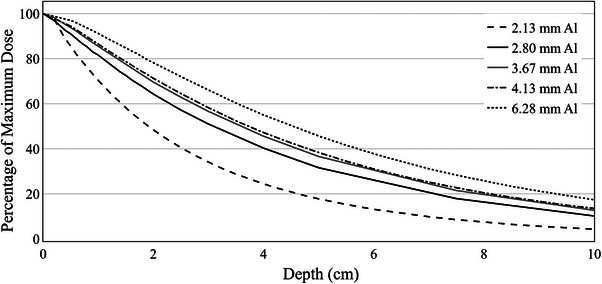
Percentage depth dose curves for four different clinical 100 kVp beams with half‐value layers (HVLs) 2.1–6.3 mm Aluminum.

To demonstrate the effect of large differences in HVL on the penetrating power of beams with identical kVp, measured percentage depth dose (PDD) curves are plotted for five clinical beam energies of 100 kVp with HVL ranging between 2.13 and 6.28 mm Aluminum (Figure 3). These PDDs were plotted using measured data provided by four departments that provided this additional information.

### 3.2 Reference dosimetry equipment

**FIGURE 4:**
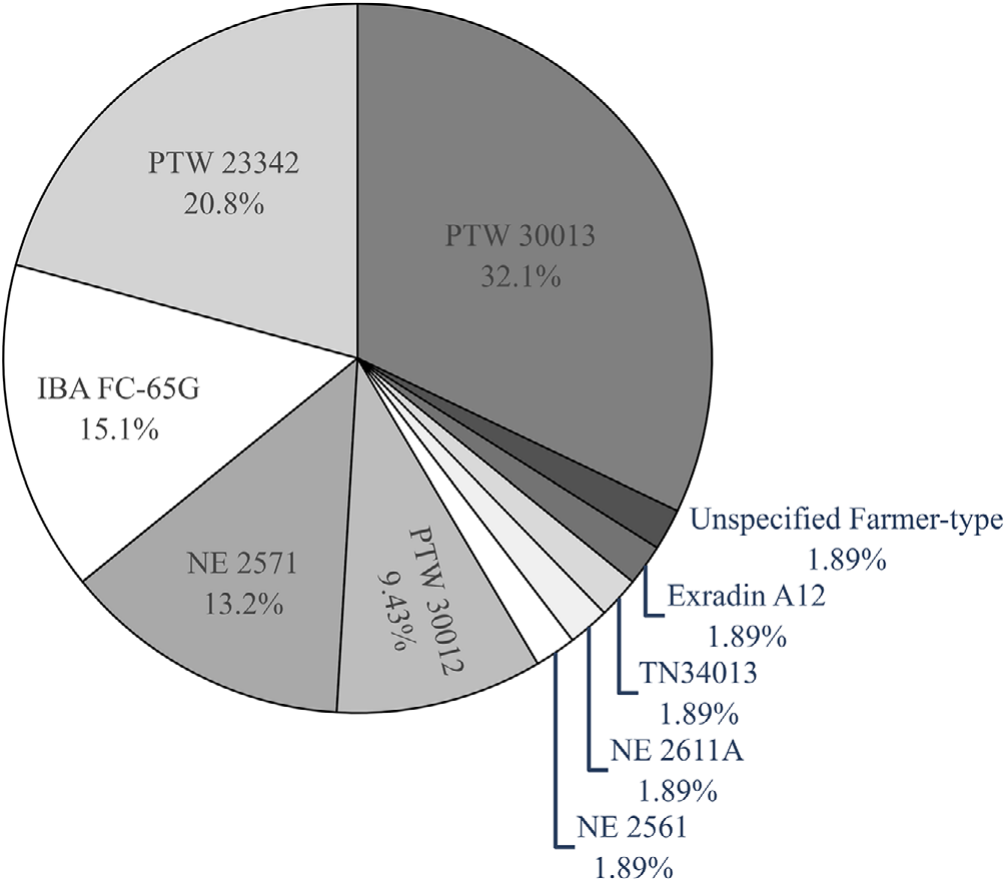
Survey reported detector types used for reference dosimetry of kilovoltage units across Australia and New Zealand.

A total of 53 ionization chambers were reported in response to question 2, with some departments having multiple detectors calibrated in terms of K_air_ and used for reference dosimetry. Figure 4 indicates the utilization of particular ionization chamber models from the cohort of departments that responded.

Of the 53 ionization chambers, 41 (77%) were Farmer‐type cylindrical ionization chambers, and 12 (23%) were soft‐x‐ray plane parallel ionization chambers. The most common cylindrical ionization chambers were the PTW30013 (42%), IBA FC65‐G (20%), and NE2571 (17%) types. The PTW 23342 was the most frequently used plane parallel ionization chamber, accounting for 92% of the soft x‐ray chambers reported.

Farmer‐type cylindrical chambers were used for absorbed dose determination of 161 reported beams, with generating potentials between 30 and 300 kVp (HVL range 1.04 mm Al–3.88 mm Cu). Forty farmer‐type chambers were calibrated in the ARPANSA MEX beam set, 1 was calibrated in the ARPANSA LEX beam set. All soft‐x‐ray chambers were reported to have been calibrated in the ARPANSA LEX beam calibration set and were used for 24 of the therapeutic beams (≤100 kVp, HVL range 0.08–4.02 mm Al).

### 3.3 Air KERMA calibration factor determination

**FIGURE 5:**
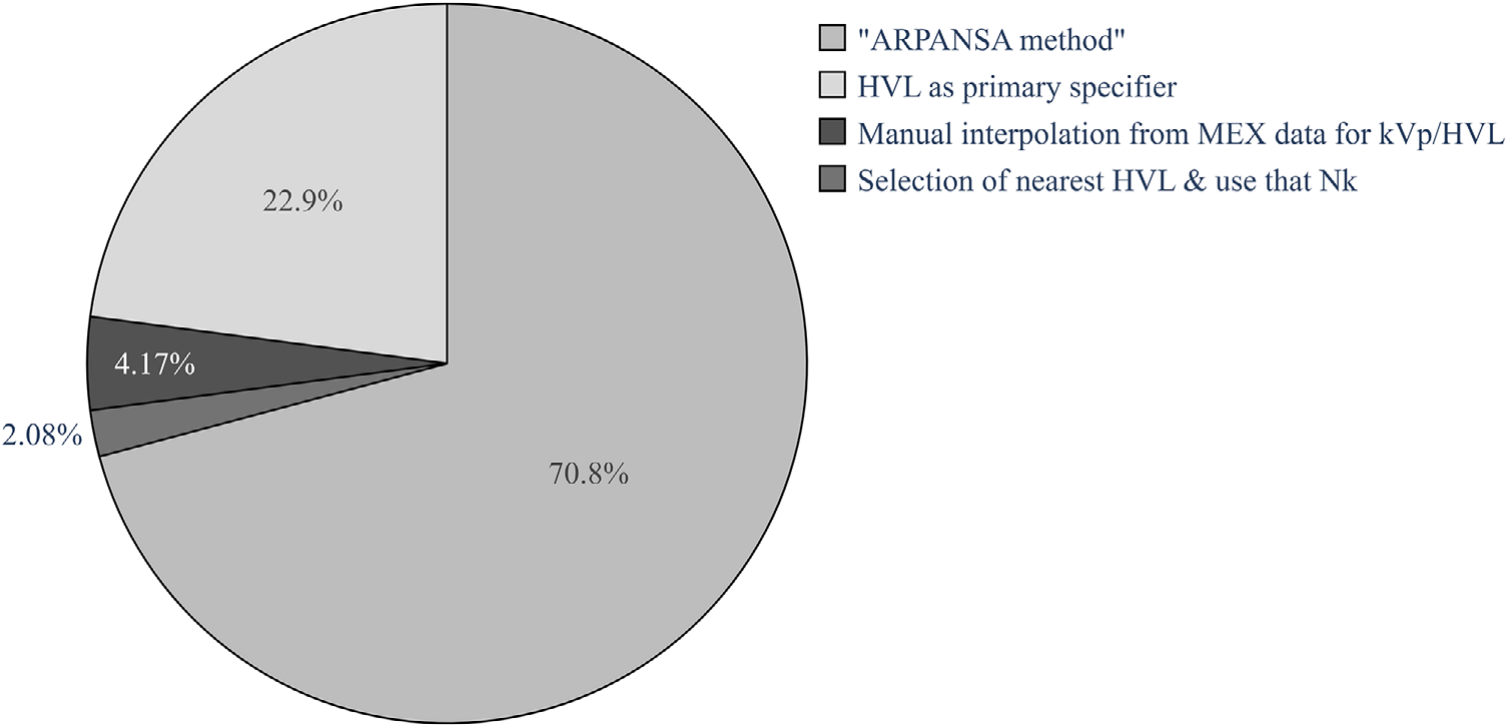
Reported method of ionization chamber air kerma calibration factor (N_K_) determination from calibration beam quality to the clinical beam quality across Australia and New Zealand.

In reply to question 3 regarding the method of N_K_ determination for reference chambers, 48 responses were received, with some departments utilizing multiple methods depending on the beam quality and equipment. Responses for question 3 are summarized in Figure 5.

The draft ARPANSA document is the most common method utilized for interpolation of MEX N_K_ data across Australia and New Zealand, with 34 respondents (71%) utilizing it. Interpolation based on beam HVL alone is the second most common method, with 11 respondents (23%) utilizing it. Three departments (6%) utilize other methods to determine N_K_ from the ARPANSA MEX data based on the assessment of their chamber response in the MEX calibration beam set.

Of the respondents using the ARPANSA draft document, 33 (97%) were able to provide detailed information of the utilization of methods within the document for their clinical beams, as shown in Table 2. Due to the range of clinical beam qualities and those provided by ARPANSA, multiple cases were frequently used in a single department to determine N_K_. Departments routinely use Case 1; with Case 2, Case 3, and Case 4 utilized when necessary. It must be noted that the methods for Case 3 vary based on the HVL of the user beam.

**TABLE 2:** Frequency of utilization of different cases to obtain the air kerma calibration factor (N_K_) in the clinical beam quality for ionization chambers calibrated in the ARPANSA MEX beam set as reported in the survey across Australia and New Zealand.

ARPANSA draft document case utilization	Frequency
Case 1	33
Case 2	8
Case 3	15
Case 4	1
Non‐routine use of Case	3

### 3.4 Clinical utilization

**FIGURE 6:**
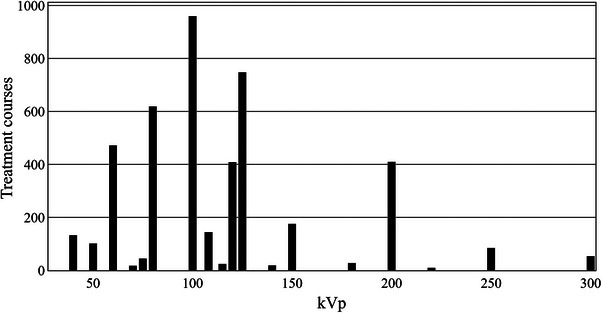
Number of treatment courses as a function of kVp in the last 5 years reported in the survey for 18 clinical kV treatment units in Australia and New Zealand.

In response to question 4, 18 departments (42% of the departments having kV units) were able to provide data on the clinical utilization of their kV units, with a total of 4458 treatment courses with their corresponding treatment kVp reported. Figure 6 is a frequency histogram of the number of treatment courses for a specific beam kVp.

## 4 DISCUSSION

Across Australian and New Zealand departments, of the 185 clinical beams reported, 100 kVp is the most common clinical beam energy. Results were similar to a 2016 survey on the status of kV radiotherapy in the United Kingdom reporting a median energy of 120 kVp.^11^ A 2009 survey of radiotherapy utilization in Australia^12^ graphically presented the distribution of kVp and HVL for 26 kV units, with the energy range 100–120 kVp appearing to be the median energy. There has been an increase of 17 kV units across Australia, however, the number of beams has only increased by two, with 183 beams reported in 2009.^12^ This suggests that although there has been an increase in the number of kV treatment units, these units are operating with far fewer clinical beams. Anecdotally, departmental multidisciplinary decisions on the clinical use of different energies and ongoing maintenance and quality assurance of a large number of beams per unit have been the cause of this reduction.

The 2009 study also reported that the reference beams at ARPANSA did not cover the full range of beams in clinical use and that ARPANSA would investigate changing the calibration dataset. This did occur in 2017, with the introduction of the MEX calibration beam set.^12^

Figure 3 has demonstrated that the dose deposition as a function of depth is not equivalent between beams of the same kVp. Given the large range of beam HVL for a single kVp shown in Figure 2, it must be kept in mind when examining the literature, of the differences that can exist between beams of the same nominal kVp. Clinically, the radiation oncologist must be fully aware of the dose deposition characteristics for the clinical beam energy in their department to ensure target volumes are being covered adequately and organs at risk are effectively spared.

Equipment used for reference dosimetry complied with recommendations by the ACPSEM^4^ and AAPM TG61,^8^ with only “Farmer‐type” cylindrical ionization chambers and thin‐window plane parallel chambers being utilized for absorbed dose determination. Analysis of the reference dosimetry equipment used for each beam kVp was possible based on the survey data. It was identified that 17 out of 38 beams with ≤70 kVp had reference dosimetry performed using cylindrical chambers, which does not adhere to recommendations from the ACPSEM^4^ and AAPM TG61.^8^ Twenty‐two out of 73 beams between 70 and 100 kVp had reference dosimetry performed using cylindrical ionization chambers, which is acceptable in the ACPSEM recommendations^4^ provided the variation in N_K_ is less than 3% over the range of 70–300 kVp. The AAPM TG61 report^8^ does not endorse the use of cylindrical chambers in beams <100 kVp. Without obtaining detailed calibration reports for each of the ionization chambers used in beams <100 kVp the authors cannot conclude that the chambers are not suitable for use; it is assumed that the local medical physicist has undertaken an evaluation of chamber response in the clinical range of kVp and made an informed decision regarding suitability.

Each standards laboratory has its own unique beam set used for calibration, with different kVp and range of HVL. The method of recommended interpolation will differ based on the beam qualities offered by the standards laboratory. At present, the document circulating from ARPANSA is in draft format and is not mandated for use, nor is it published. The survey has shown that departments are using varied approaches for the determination of ionization chamber N_K_, which can vary the resulting N_K_ by up to 1%. This directly impacts the determination of the absorbed dose for the kV beam, and hence dose delivered to the patient. A consistent method of interpolation for departments utilizing ionization chambers calibrated in a reference beam set at the standards laboratory would ensure consistency in absorbed dose determination for kV units.

Clinical utilization numbers proved difficult to obtain. One of the main issues respondents had, was being able to extract treatment information easily from the Oncology Information System (OIS), with two of the 18 departments providing an estimate of the number of patients treated based on other data extraction methods, or simply a total number of patient courses treated with kV without breaking down further into individual treatment energies.

Data relating to treatment courses is biased by the following factors; clinical caseloads in a department, radiation oncologist treatment preferences, clinical kVp available for use, and the years of service for a kV unit. It was noted that some kV units were clinical for less than 5 years, hence patient treatment course numbers were lower than other departments with established kV units.

## 5 CONCLUSION

The results of this survey have provided an estimate of the current clinical utilization and number of kV units across Australia and New Zealand.

All departments surveyed are using ACPSEM and AAPM TG61 recommended equipment for reference dosimetry, however, utilization of the equipment for absorbed dose determination does not comply with recommendations in all cases. Methods of N_K_ determination for ionization chambers calibrated in medium‐energy x‐ray beams at the standards laboratory also varied, however, most departments were utilizing methods outlined in a draft document from the primary standards laboratory.

The data presented can be used as a point of reference for future investigations into clinical utilization and reference dosimetry methods across Australia and New Zealand or for comparisons with other countries.

## AUTHOR CONTRIBUTIONS

Madelaine Tyler has led the project, designing the survey, corresponding with departments, collating, and analyzing results. Mitchell Duncan has supported the project in all aspects, provided consultation, and assisted with data analysis. Joanne McNamara has supported the project in all aspects, provided consultation, and assisted with data analysis.

## CONFLICT OF INTEREST STATEMENT

The authors declare no conflicts of interest.
